# Malignant Melanoma Metastasizes to Colonic Polyp

**DOI:** 10.7759/cureus.2822

**Published:** 2018-06-18

**Authors:** Muhammad Azharuddin, Ahmad Sharayah, Syed H Abbas, Kenneth Belitsis

**Affiliations:** 1 Internal Medicine, Monmouth Medical Center, Long Branch, USA; 2 Pathology, Monmouth Medical Center, Long Branch, USA; 3 Gastroenterology, Monmouth Medical Center, Long Branch, USA

**Keywords:** metastasis, malignancy, colonic polyp, immunotherapy, gastrointestinal metastasis, colonoscopy

## Abstract

Malignant tumors metastasizing to the colon has been observed rarely. Gastrointestinal metastasis can present as benign, unpigmented polyps endoscopically. Most patients do not display any symptoms, and if symptomatic, they usually present with gastrointestinal bleeding. For patients with the history of melanoma, histopathology of polyp can change or alter the course of management. This is a case of a 74-year-old male diagnosed with recurrent melanoma of left ear. Colon cancer screening found blood in his stool. Colonoscopy displayed to have three polyps, one polyp was found to be malignant melanoma. The patient was started on Pembrolizumab, and was tolerating immunotherapy well with no new complaints three months later.

## Introduction

Malignant tumor metastasizing to the colon has been observed to be extremely rare. Although rare, malignant melanoma was believed to be the most common tumor metastasizing to the colon [1-2]. It endoscopically can imitate simple polyps [1]. However, breast cancer was recently reported to be the top cause for colonic metastases. Gastrointestinal (GI) malignancies constitute only 1-3% of all malignant melanomas [2].

Melanoma metastasizing to the GI tract usually affects the distal small intestine, followed by the stomach, rectum, and colon. Metastasis to the GI tract may present as anemia, bleeding, weight loss, abdominal pain, or bowel obstruction [2]. Most patients do not display any symptoms, and have a primary lesion. Patients showing symptoms usually have a poorer prognosis [1, 3].

## Case presentation

A 74-year-old Caucasian male had a past medical history of hypertension and gout. He was also diagnosed with melanoma of left ear at left medial antihelix seven years ago, and it was stage IIB and treated with wide local excision and sentinel lymph node excision. Two years later, he developed recurrent melanoma of left ear, and it was staged IIIB treated with left aurilectomy. He underwent colon cancer screening. His stool was positive for blood. He underwent colonoscopy and was found to have three polyps. One polyp was 1 cm in ascending colon, 1 cm polyp in the sigmoid colon, and 4.5 cm polyp in sigmoid colon. Differential diagnosis was benign polyp, and primary colon cancer.

**Figure 1:**
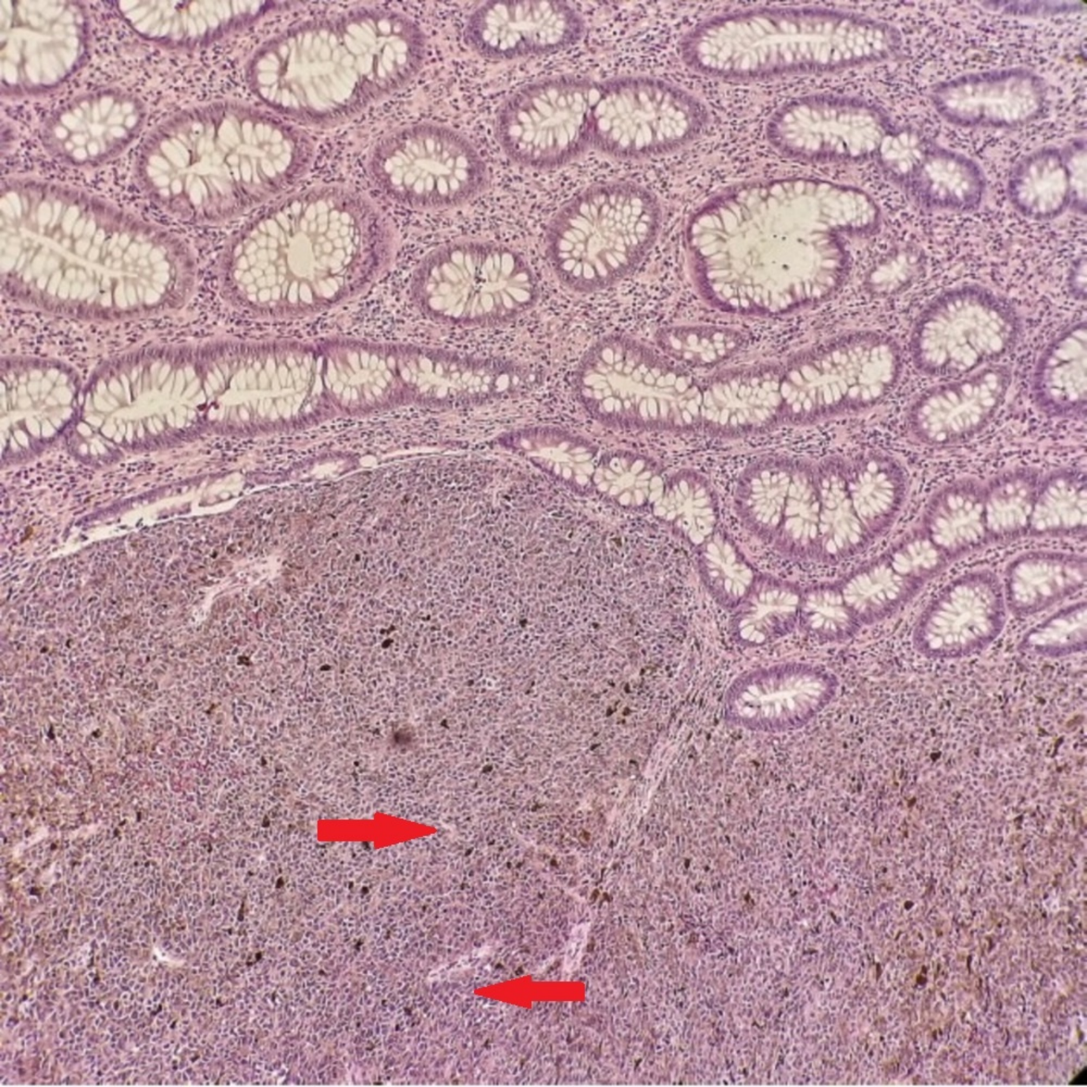
Haematoxylin and eosin. Normal colonic mucosa with tumor showing discohesive cells with pink cytoplasm, pleomorphic nuclei, macronuclei, and pigmentation. Arrow indicates pleomorphic nuclei.

**Figure 2:**
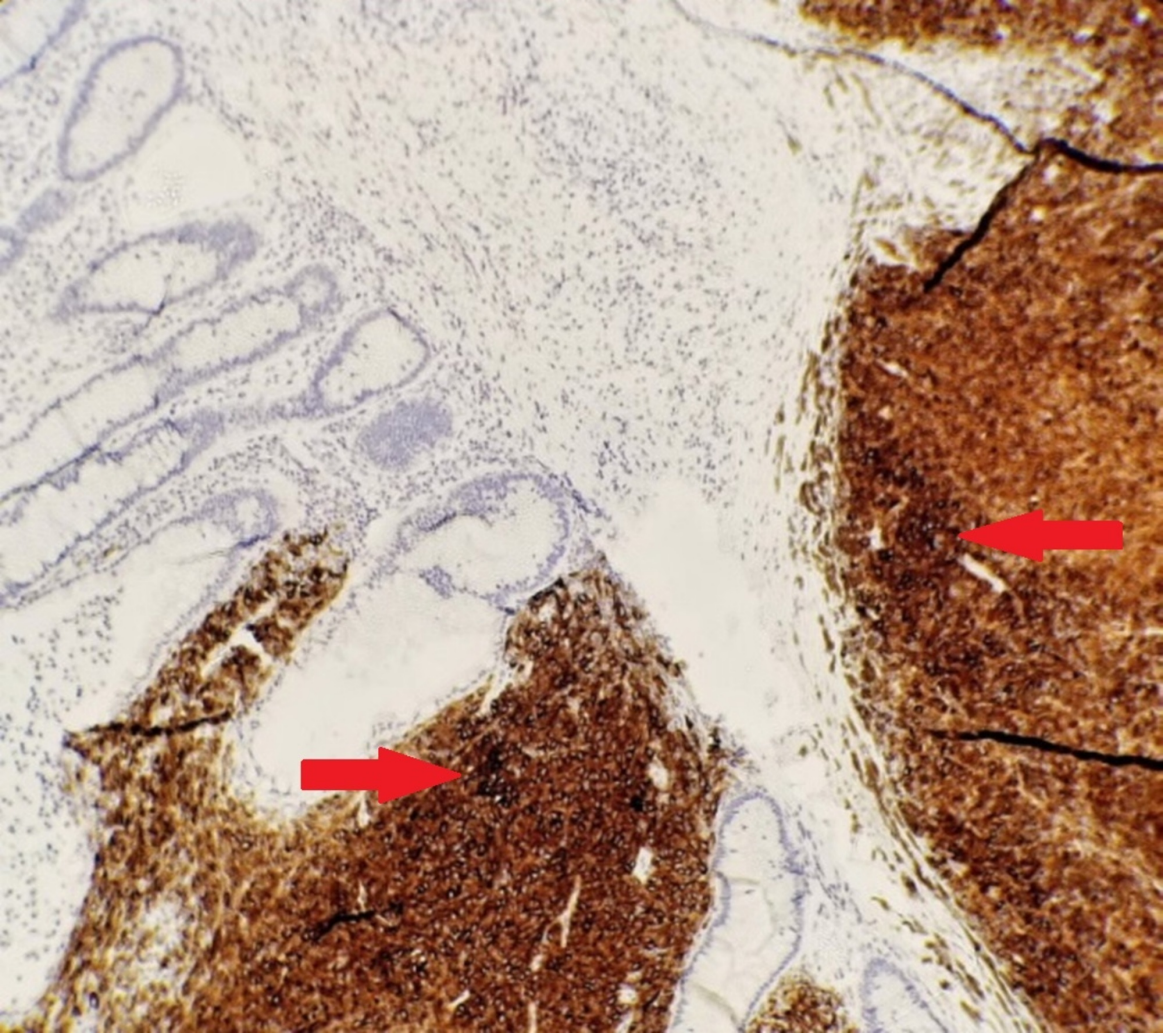
Melana stained slide. Staining of the tumor cells. Normal colonic mucosa that is unstained can also be seen.

Biopsy displayed malignant melanoma in the largest polyp in sigmoid colon with negative margins. Tumor cells were positive for melanin A and negative for MCK. Histopathology confirmed malignant melanoma in sigmoid colonic polyp (Figures 1, 2). Molecular analysis showed NRAS Q61R mutation (NRAS is in the Ras family of oncogenes), B2M copy number loss. Other two polyps showed tubular adenoma. All polyps were resected. His previous colonoscopy 12 years ago was normal. Endoscopy did not reveal any polyp in the stomach or small intestine.

Positron emission tomography or computed tomography (PET/CT) exhibited increased fludeoxyglucose avidity in right adrenal gland, gallbladder, and in right orbital apex avidity. It also showed hyper-metabolic ac nodule in the left orbital apex, and no recurrence at the left auricular region. Hyper-metabolic activity was seen in gallbladder of the size of 1.57 cm and right adrenal of 1.8 x 1.2 cm. Magnetic resonance imaging of the brain showed no evidence of metastases.

The patient was started on pembrolizumab. He was closely followed up with the oncologist. Repeated PET/CT displayed stable activity in right adrenal gland, gallbladder, and right orbital apex. The patient was tolerating immunotherapy well, and he has no new complaint during follow-up three months later.

## Discussion

Cutaneous malignant melanoma is a common type of tumor that metastasizes to the GI tract. However, most patients with GI metastatic melanoma do not display symptoms, and only about 4.4% are diagnosed before death [1]. In 50% of cases, bleeding (occult or overt) was the most common presenting symptom [2]. Due to the asymptomatic nature, only a few patients initially present with metastatic GI malignant melanoma [1]. A retrospective study of 2500 malignant melanoma patients resulted in 110 (4.4%) having pre-mortem GI metastatic disease. Out of the 100 living patients, only 22 had colonic metastases [1, 4]. The most frequent sites for metastasis were found to be the small intestine (35%), colon (15.5%), and stomach (7%) [1]. Our case is of interest as the patient was symptomatic and presented with polyps infiltrated by malignant melanoma [1].

Guaiac fecal occult blood test (G-FOBT) screening performed once a year lowers deaths by colorectal cancer (CRC) by more than 16–33%. G-FOBT is preferred since it is private, non-invasive, and low cost. However, G-FOBT has disadvantages including low sensitivity for CRC and advanced adenomatous polyp, interval testing, and possibly false assessment [5]. The gold standard for colon cancer screening is colonoscopy. In our patient, cologuard test was positive for blood, thereby leading to other invasive investigation colonoscopy and endoscopy. Polyps found in the histology on the sigmoid colon showed melanoma.

In metastasis to colon, there are various presentations found in endoscopy including colon intussusception, multiple colon polyps, and fungating masses resembling primary colon cancer [2]. Although it is very difficult to decipher between primary metastasis and metastatic melanoma of GI tract, there are some protocols created to differentiate between the two [2]. Diagnostic factors for primary melanoma involve not having current or formerly removed melanoma, irregular increase of skin melanocytes, no other organ involvement, and in situ lesion located in the overlying or nearby GI epithelium [6]. Our patient has the history of melanoma and its recurrence and detailed workup also showed lesion in orbital apex, gallbladder, and right adrenal gland.

Primary melanoma is typically one lesion without metastasis to other organ involvement. Compared to skin melanomas, primary or metastatic malignant melanomas of the gastrointestinal tract, either primary or metastatic, have lower prognosis and are more violent, with an average of only 4-6 months of survival. Patients with metastatic melanoma typically have a prognosis of less than 10 months of survival, especially those presenting with bowel obstruction, perforation, and peritonitis [2].

Immunotherapy with ipilimumab (anti-CTLA4) or BRAF inhibitors, a human gene that encodes a protein called B-Raf, can increase survival rate in selected patients [2]. Every patient with metastatic disease should be tested for BRAF V600E mutation for the possibility to being treated with BRAF inhibitors therapy of vemurafenib or sorafenib [2]. A few cases were reported of colonic polyps developing from BRAF inhibitor treatment of melanoma. This may be due to inconsistent mitogen-activated protein-kinase activation [7]. Immunotherapy with interleukin 2 may boost survival rate in selected patients, but can also be toxic [2].

Surgery may serve a key role as a symptomatic measure and may increase survival. Stage of tumor during the disease may be integral in guiding surgery, especially in cases of an isolated metastatic lesion or where several lesions are in proximity. However, surgical excision may be impossible in cases of multiple lesions. Patients treated surgically are found to live significantly longer compared to patients treated without surgery [2]. In cases of advanced metastatic melanoma, cytotoxic chemotherapy with single-agent or combination therapy has not been found to be beneficial [2].

## Conclusions

Malignant tumors metastasizing to the colon are unusual. This patient underwent colonoscopy, in which one polyp was found to be malignant melanoma. He underwent immunotherapy, and had no new complaints three months later. Metastatic melanoma to the gastrointestinal tract has a very poor prognosis. Very few cases have been reported with colonic polyp on colorectal screening diagnosed with metastatic melanoma.
